# Moderate-Low Risk Breast Cancer Gene Expression in a Romanian Population

**DOI:** 10.3390/ijms26115313

**Published:** 2025-05-31

**Authors:** Iulian Gabriel Goidescu, Ioana Cristina Rotar, Georgiana Nemeti, Adelina Staicu, Mihai Surcel, Gheorghe Cruciat, Daniel Mureșan, Cerasela Goidescu, Dan Eniu

**Affiliations:** 1 Obstetrics and Gynecology I, Mother and Child Department, University of Medicine and Pharmacy “Iuliu Hatieganu”, 400006 Cluj-Napoca, Romania; goidescu.iulian@elearn.umfcluj.ro (I.G.G.); georgiana.nemeti@elearn.umfcluj.ro (G.N.); adelina.staicu@elearn.umfcluj.ro (A.S.); mihai.surcel@elearn.umfcluj.ro (M.S.); gheorghe.cruciat@elearn.umfcluj.ro (G.C.); muresandaniel01@elearn.umfcluj.ro (D.M.); 2 Department of Internal Medicine, Medical Clinic I—Internal Medicine, Cardiology and Gastroenterology, University of Medicine and Pharmacy “Iuliu Hatieganu”, 400006 Cluj-Napoca, Romania; sava.cerasela@elearn.umfcluj.ro; 3 Department of Surgery 2, University Emergency Hospital, University of Medicine and Pharmacy “Iuliu Hatieganu”, 400006 Cluj-Napoca, Romania; daneniu@elearn.umfcluj.ro

**Keywords:** *ATM*, *CHEK2*, *BARD1*, low penetrance genes, Lynch syndrome

## Abstract

Multigene panel testing for hereditary breast and ovarian cancer is becoming a standard in medical care. Recent studies highlight the importance of pathogenic variants in genes with moderate or low penetrance. 255 consecutive breast cancer cases who met the criteria for genetic testing were approached by next-generation sequencing. From 104 pathogenic mutations identified, 21 were in moderate-risk genes, three in low-risk genes and eight in the group with insufficient evidence genes. The most frequent PVs in moderate-risk genes were in the *CHEK2 gene—Checkpoint kinase 2 gene* (13 cases), the *ATM* gene—*Ataxia-telangiectasia Mutated gene* (six cases), *BARD1—BRCA1-associated ring domain 1 gene* (one case) and *RAD 51C–radiation sensitive 51 Paralog C*—(one case) genes. Among the low-risk genes, we identified only three pathogenic mutations (two in *MSH1* gene—melanocyte-stimulating hormone gene—and one in *MLH1* gene—*MutL homolog 1* gene). Reporting on low-risk mutations and those with insufficient evidence regarding breast cancer risk is valuable to enable a more comprehensive view of genetic factors influencing disease development and improve screening protocols, tailor diagnostic strategies, and individualize treatment plans. This approach also enhances our understanding of BC risk in various populations, potentially leading to new insights into genetic contributions to cancer and the refinement of risk models for patient care.

## 1. Introduction

Breast Cancer (BC) is the most common cancer type in women worldwide and a leading cause of death [1], with an estimated 10–20% rate of hereditary cases [2]. The background for developing hereditary breast and ovarian cancer (HBOC) is the occurrence of germline mutations in susceptibility genes and their interplay with lifestyle factors (diet, physical activity, smoking, alcohol consumption), exposure to environmental toxins, and reproductive history (age at menarche, parity, breastfeeding, and age at menopause). This results in individual risk profiles regarding age at BC onset/diagnosis and disease severity/stage/evolutive potential [3].

The NCCN (National Comprehensive Cancer Network) and ESO-ESMO (European School of Oncology, European Society for Medical Oncology) guidelines support genetic testing to personalize risk stratification and tailor medical systemic treatment in BC [4]. Germline genetic testing for women with BC is nowadays a well-established part of clinical practice. A standardized gene variant nomenclature is maintained and constantly updated by the Human Genome Variation Society (HGVS).

Physicians should consider integrating genetic information into treatment decisions and follow-up of BC patients and their offspring.

BC susceptibility genes can be grouped into two classes: high- and low-penetrance genes (low risk or insufficient evidence to make any recommendations for patients) [5]. At the beginning of the genetic-guided approach, clinicians primarily focused on high-risk genes, such as *BRCA1/2—BReast Cancer 1/2, TP53—tumor protein p53, PALB2 Partner and Localizer of BRCA2-, CDH1—Cadherin 1, STK11—serine/threonine kinase 11 and PTEN—Phosphatase and TENsin homolog deleted on chromosome 10*.

For many genes besides BRCA1/2, there is limited information on the normal range of genetic variation, resulting in the identification of numerous variants of unknown significance, especially in patient groups of non-European ancestry [5]. For these reasons, the NCCN guideline emphasizes two key points: (1) variants of unknown significance (VUSs) should not influence medical management decisions, and (2) for individuals carrying a VUS, family history should guide risk reduction and screening strategies [5]. Furthermore, a substaging of these VUS mutations has been proposed in suspicious VUSs, clinically relevant VUSs, or nonclinically relevant VUSs, to emphasize that management is based on the patient’s personal and family history and the interpretation of the variant [6].

However, recent studies are beginning to highlight the importance of pathogenic variants (PVs) in genes with moderate or low penetrance, particularly in imaging diagnosis and the natural progression of certain forms of BC in patients carrying PVs in specific genes, as well as their varied responses to adjuvant treatments in specific cases [7,8,9].

For example, it is known that the loss of function of the *ATM-CHEK2-*p53 cascade (*Ataxia-telangiectasia Mutated/Checkpoint kinase 2—*tumor protein p53) is associated with resistance to anthracycline/mitomycin-containing chemotherapy in BC patients [7], or that the ATM carrier patients are more likely to develop subcutaneous necrosis and contralateral breast cancer after radiotherapy [10]. These potential complications can pose challenges, particularly for patients seeking immediate breast reconstruction, making it essential to plan plastic surgery interventions in advance to prevent such issues [11].

Two recent studies have shown that PVs in moderate-risk genes (*ATM, CHEK2, RAD51C* and *RAD51D*—*Radiation Sensitive paralog C and D*) have a significant association with BC risk, especially *ATM* and *CHEK 2* mutations (odds ratios ranging from 2.1 to 2.5) [12,13].

Furthermore, germline pathogenic/likely pathogenic (P/LP) variants in cancer predisposition genes have shown a dose effect, resulting in a more severe phenotype in homozygous than heterozygous cases and for *CHEK2* P/LP variants, the homozygous state confers a higher risk of BC than the heterozygous state [14,15]. Also, there are particular P/LP variants in moderate penetration genes that confer similar risks of BC as high-risk genes, for instance, the missense pathogenic variant c.7271T>G in *ATM* may considerably increase the risk of breast cancer in a similar proportion as P/LP variants in *BRCA2* [16].

Among the genes with a low risk of BC, the most well known are those responsible for Lynch syndrome. This hereditary cancer syndrome is caused by the heterozygous germline inactivation of the DNA mismatch repair genes *MLH1, MSH2, MSH6 and PMS2 (MutL homolog 1, melanocyte-stimulating hormone, postmeiotic segregation increased 2)*, which leads to microsatellite instability [17].

For these reasons, current guidelines also recommend identifying pathogenic variants in genes with moderate and low risk for BC, particularly since there is less global information and medical data on these genes compared to the well-studied high-risk mutations.

Our study aims to report PVs in moderate- and low-risk genes responsible for BC in a Romanian population, as well as information regarding the occurrence of PVs in genes for which there is currently insufficient or contradictory data regarding their impact on BC risk.

## 2. Results

**Figure 1:**
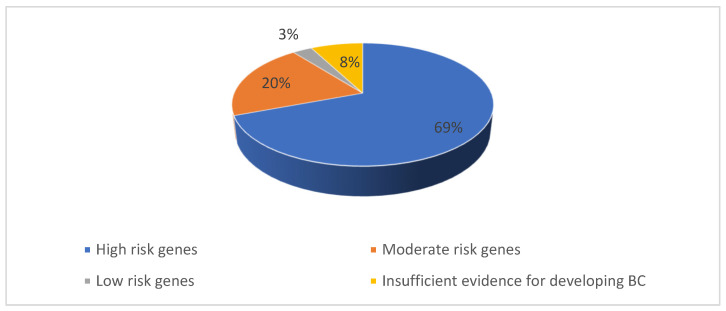
Percentage of P/LP variants identified in our study population.

**Figure 2:**
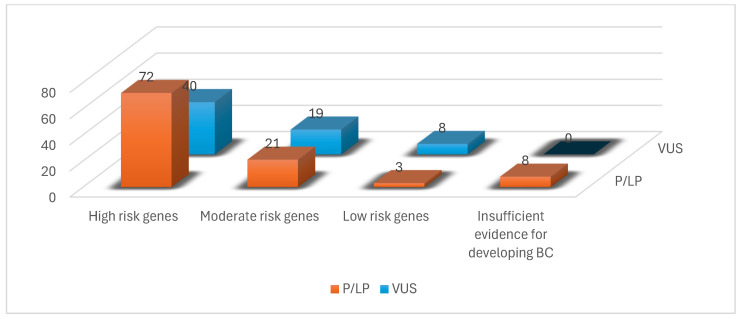
Number of P/LP variants and VUSs identified in our study population.

We identified 171 mutations in the 150 BC patient population selected for the study. 98 patients had pathogenic mutations, with a total of 104 PVs diagnosed (six patients had two pathogenic mutations each) (Figure 1). Additionally, 67 variants of uncertain significance (VUSs) were identified in 52 patients (six patients had two VUS mutations each, and nine patients from the pathogenic mutation group also had a VUS mutation) (Figure 2).

From the 104 PVs identified, most mutated genes, with 72 mutations, belonged, as expected, to the high-risk group, as shown in Figure 1. The rest of the PVs were 21 mutations in the moderate-risk genes category, three mutations in the low-risk genes category and eight mutations in the group with insufficient evidence for developing BC (Figure 2, Table 1).

The distribution of pathogenic *CHEK2* and *ATM* mutation variants identified in the study group patients; mutation type and their estimated risk are depicted in Table 2 and Table 3.

## 3. Discussion

The result of genetic testing in our patient population provided an expected majority of high- and moderate-BC-risk mutations, compared to genes with low penetrance or insufficient evidence. A sequential, detailed analysis of results focusing on each of the genetic mutations identified is provided below.

The *CHEK2* gene is located at chromosome 22q12.1, encodes a 60-kDa protein consisting of 546 amino acid residues [18] and plays an important role in the DNA-damage signaling network [19]. However, the BC risk for a woman carrier of the *CHEK2* mutation is not only dependent on the presence of the mutation itself but also on the familial history of cancers [20]. The two most frequent CHEK2 mutations in European populations are c.470T>C (p.I157T) and c.1100delC [21].

**Figure 3:**
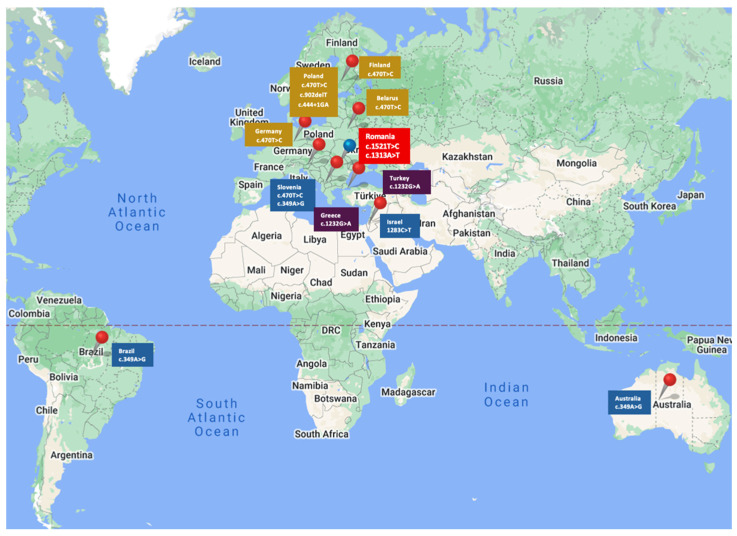
Reported spread of *CHEK2* mutation variants.

The c.470T>C variant has been reported more frequently in Europe and has been associated with BC in countries such as Poland, Finland, Germany, and Belarus (Figure 3) [22,23].

In a study conducted by Cybulski et al. in 2011, the authors identified the c.470T>C PV in 7.1% of patients with BC (535 patients) as being the most common pathogenic *CHEK2* variant in Polish people (Figure 3) [20]. The odds ratio for BC was 1.5 (95% CI, 1.2 to 1.7) in women diagnosed <50 years and 1.6 (95% CI, 1.3 to 2.0) in women diagnosed >50 years [20]. They also observed that lobular type BC was slightly more common in women with a *CHEK2* missense mutation c.470T>C than in non-carriers (18.4% vs. 13.9%, respectively; *p* = 0.009) [20]. This conclusion was not superposable on our patient population, where all seven patients with c.470T>C variants had an invasive ductal histology type.

In a 2012, a comprehensive metanalysis of 15 case-control studies conducted by Chuan Liu [24], the authors also concluded that the *CHEK2* c.470T>C variant has a 1.5-fold increased risk for BC, like previous reports. This observation was also confirmed in three of our patients diagnosed with bilateral BC: two carrying the c.470T>C variant and one carrying the c.1232G>A variant.

A more recent study conducted by Bychkovsky et al. in 2022 confirmed that the c.470T>C variant is not associated with a higher risk of BC in the absence of family history, but it raised the suspicion of its association with a higher risk of bilateral BC [25]. This observation had been previously reported by Nizic-Kos T. et al., who pointed out that bilateral BC was diagnosed in 19.5% of Slovenian BC patients with *CHEK2* PV (Figure 3) [26].

The 1283C>T (p.Ser428Phe) and c.349A>G (p.Arg117Gly) variants are rare missense mutations associated with BC in some populations. The 1283C>T (p.Ser428Phe) variant was noted in the Israelian population [27] and the c.349A>G (p.Arg117Gly) was reported in the Australian [28], Brazilian [29] and Slovenian populations (Figure 3) [26].

The c.1232G>A (p.Trp411Ter) is a nonsense mutation reported as pathogenic in the Greek and Turkish populations [30,31], whom we also signaled in a previous publication (Figure 3) [32].

Another *CHEK2* gene variant, c.909?-1095 delA, is a deletion of the genomic region encompassing exons 9 and 10, which leads to an absent or disrupted protein production, which was found in the Polish population [33]. Another rare frameshift mutation, the c.902delT (p.Leu301fs) variant, was also found and described in the Polish population (Figure 3) [34,35].

The *CHEK2* c.444+1G>A variant, also reported as IVS2+1G>A, occurs in a splice site (donor) and is, therefore, predicted to disrupt or distort the normal gene production. The c.444+1G>A variant has been shown to be one of three *CHEK2* founder variants in the Polish population [22,35]. Bychkovsky et al. demonstrated that BC prevalence was highest among participants with *CHEK 2* c.444+1G>A variant (OR, 2.63; 95% CI, 1.59–4.35; *p* < 0.001) [25].

The *ATM* gene encodes a kinase that plays a critical role in DNA double-strand break repair pathways and cellular response to DNA damage. Heterozygosity for loss-of-function variants in the *ATM* gene and truncating or missense variants has been associated with moderately increased BC risk, which can build up to a relative risk (RR) of 2.8% [36].

Studies have shown that loss-of-function (LoF) variants in the *ATM* gene significantly increase BC risk compared to missense variants [37]. LoF mutations, such as nonsense or frameshift mutations, lead to a complete loss of protein function, which more drastically impairs the DNA damage repair mechanism. This impairment results in a much higher predisposition to cancer, with risk levels up to 10 times greater than those associated with missense variants, which typically only cause partial dysfunction rather than a complete loss of protein functionality [37].

In the Romanian population, we identified six PV in the *ATM* gene, two cases of c.7630-2A>C variant, both being diagnosed in young patients with BC, and one case associated with a PV in the *CHEK2* gene (c.444+1G>A).

We had also reported encountering the c.2250G>A variant in our previous research [32] and had confirmed it as a PV in a recent study from 2020 [38].

The three frameshift variants in the *ATM* gene identified in our study (c.1564_1565delGA, c.5318delA, c.6628delC) have been confirmed as PVs linked with BC by previous authors [38,39].

Interestingly, the patient with the PV c.5318delA in the *ATM* gene also had a PV in the *MUTYH* gene (c.721C>T) and two VUS mutations in the *ATM* (c.680C>T) and BRCA2 (c. 6626T>C) genes.

The *BARD1* gene (BRCA1-associated ring domain 1) is located on chromosome 2 (2q34-35), and the protein it synthesizes plays a role in the onco-suppressive effect of the *BRCA1-BARD1* complex. At the same time, the *BARD1* gene has been involved in the regulation of cell apoptosis (*BARD1* promotes the formation of p53 and DNA-PK complexes, which then allows for p53 phosphorylation by *ATM*) and interreacts with *MSH2* and *MLH2*, two important mismatch repair proteins [40]. For all these reasons, the *BARD1* gene is now considered to play an important role in hereditary BC risk, and it has been postulated that it should be included in multi-panel HBOC tests [41].

The *BARD 1* c.632T>A variant was also identified in a previous study in the Romanian population, alongside two other PVs, accounting for 3% of BC patients with pathogenic mutations [42].

The Breast Cancer Association Consortium and the CARRIERS case-control studies also found associations between PV in the *BARD1* gene and an increased risk of developing triple-negative breast cancer, something we had also reported in our patients [12].

Furthermore, the presence of PV in the *BARD*1 gene introduces the possibility of incorporating PARP inhibitors (Poly (ADP-ribose) polymerase inhibitors) as therapeutic agents, patients benefit from this treatment that originally targeted other forms of HBOC with pathogenic mutations in genes such as *BRCA1* and *PALB2* [43].

When it comes to the *RAD51* gene, it has been found to interact directly with *BRCA2*, which mediates *RAD51* polymerization at sites of double strand breaks, and the *RAD51C* and *RAD51D* mutations are essential for homologous recombination activity [44], alongside three other paralogs: *RAD51B, Xrcc2, and Xrcc3* (*X-ray repair cross-complementing 2 and 3, respectively*) [45].

The *RAD51C* c.905-2A>G is a splice acceptor variant, considered to be likely pathogenic, and it was confirmed in a triple-negative breast cancer patient who also had a VUS in the *MUTYH* gene (c.536A>G).

In the category of genes with a low risk of BC, the most important are those connected to Lynch syndrome. Lynch syndrome (formerly known as the hereditary nonpolyposis colorectal cancer syndrome) represents an autosomal dominantly inherited cancer predisposition accounting for 2–5% of the total colorectal cancers. Seventy percent of Lynch syndrome cases result from a germline P/LP variant in one of four DNA mismatch repair genes (*MLH1, MSH2, MSH6, or PMS2*) [46,47]. From the germline mutations of these four genes responsible for colorectal cancer, the most frequent are encountered in the *MLH1* and *MSH2* genes (50% and 40%, respectively, of all mutations), with the remaining 10% of mutations being present in the *MSH 6* and *PMS2* genes [48].

Predominantly, Lynch syndrome is associated with an increased risk of colorectal cancer, but carriers of PVs have an increased carcinogenic risk for endometrial and ovarian cancer, and, in recent years, numerous studies have attributed an increased risk of these patients for developing BC as well [48,49,50].

Studies evaluating BC risk in women with Lynch syndrome have been conflicting, some authors reporting an up to fourfold increased risk of BC, while others found no risk increase. This situation may be caused by previous studies reporting BC risk in populations selected on the grounds of having colorectal cancer, and usually these cohorts consisted only of *MLH1* and *MSH2* carriers (which account for approximately 90% of the PVs associated with colorectal cancer) and few *MSH6* and *PMS2* carriers [48,51]. We may thus speculate that the colorectal cancer-targeted selection of patients led to a certain genetic mutation mapping, which is not appropriate for evaluating the relationship between Lynch syndrome and breast cancer. Although the risk of hereditary BC is <15% in patients with Lynch syndrome, the NCCN guidelines recommend BC testing, especially in patients with a positive family history [50].

In a study published by Nicole Buerki et al. in 2012, the authors concluded that the cumulative risk for developing BC in *MLH1/MSH2* carriers was 5.2% until age 70 years, with a higher risk for *MSH2* PV carriers, which can reach up to 10.1% [48]. However, in a later study from 2015, the authors reported a higher risk of BC in *MLH1* PV carriers of up to 18.6% and only a 11.2% for *MSH2* carriers, concluding that women with PVs in *MLH1* and *MSH2* genes should be considered for breast screening at earlier ages than those recommended by national screening programs [52].

A recent analysis conducted retrospectively on more than 50,000 women tested with multigene panels for HBOC observed a higher risk of BC in carriers of *MSH6* and *PMS2* mutations compared to older studies, which focused on identifying the risk of BC in patient cohorts selected on account of having colorectal cancer. No association was observed for the *MLH1* or *MSH2* genes [51].

In our study, we identified only three low-risk PVs associated with Lynch syndrome. c.3261dupC is a PV in the *MSH6* gene, a duplication responsible primarily for colorectal cancer risk [53], while the c.2136delG variant is a frameshift mutation cited in the literature as also being associated with BC, but apparently without related microsatellite instability [48].

It is interesting that both our patients with pathogenic mutations in the *MSH6* gene were diagnosed with multifocal forms of BC, and the patient with the c.2136delG variant had a secondary pathogenic mutation in the *MUTYH* (*mutY homolog*) gene (c.1187G>A).

*MLH1* c.2041G>A mutation is a DNA mismatch repair gene, which was first reported in Poland as a potential founder variant responsible for colorectal cancer [54], and was then identified in the Scottish and German populations [55,56].

Regarding the insufficient evidence risk mutation group, we have identified eight PVs in the *NBN (nibrin), MUTYH* and *RAD 50* genes in the Romanian population from our study.

The NBN gene provides instructions for synthetizing a protein called nibrin involved in several critical cellular functions, including the repair of damaged DNA. Nibrin interacts with two other proteins, produced from the *MRE11A (meiotic recombination 11 homolog A)* and *RAD50* genes, to create a protein complex, which in turn interacts with the *ATM* gene-encoded protein. The result of this protein interplay is the recognition of broken DNA strands, coordinating their repair and thus maintaining the stability of the cell’s genetic information [57].

The PV we identified in four cases (c.657_661delACAAA) is a frameshift variant reported as a founder mutation in the Slavic/Eastern European population [57]. One case also depicted a secondary VUS mutation in the *MRE11A* gene (c.1091G>A).

Several published studies support the association of the c.657_661delACAAA PV with increased risk for prostate cancer [58] and lymphoid malignancies [59], but there is conflicting evidence regarding BC association [60,61,62].

There are authors who suggest an association of the PV in the *NBN* gene with BC risk [59,61]; however, a large study from 2018 conducted on 5589 patients tested negative for *BRCA1/2* mutations failed to confirm *NBN* as a BC predisposition gene [62].

The *MUTYH* gene acts as an oncosuppressor and encodes a protein involved in the base excision repair mechanism, the PV in this gene being responsible for autosomal recessive familial adenomatous polyposis type 2 [63]. The *MUTYH* gene is recessive, and either two biallelic variants or one homozygous variant are required to inactivate its function; however, there are studies suggesting that monoallelic or heterozygous variants in the *MUTYH* gene may increase cancer risk [63,64].

Because multigene panel testing for HBOC has been used on a larger scale in the last decade, many authors reported an increasing number of heterozygous germline PV in the MUTYH gene, making the association with BC even more difficult [63,64].

We identified three missense variants in Romanian patients that were also reported in a recent study in BC patients [63]; similar to their results, we observed that all these patients presented additional mutations in other genes. The patient diagnosed with the c.721C>T variant had additional mutations (one PV in *ATM* gene, and two VUSs in *ATM* and *BRCA2* genes) and the c.536A>G PV carrier also had a PV in the *RAD51C* gene (c.905-2A>G) as we have previous mentioned [65]. The third case had a c.1187G>A PV and a PV in the *MSH6* gene (c.2136delG).

The RAD50 gene encodes a protein involved in DNA double-strand break repair alongside other proteins encoded by the *MRE11* and *NBN* genes [66]. There is conflicting data regarding the penetrance and clinical significance of RAD50 germline PVs and BC risk; there is very little likely association, apparently the attention of current studies is being directed especially on the somatic PVs of the RAD 50 gene, implicated in modulation prognosis and sensitivity to platinum drugs and PARP1 [67]. We identified only one PV of the RAD50 gene (c.2165dupA), which is a frameshift variant, and three VUS variants (c.785T>G, c.1663A>G, c900G>A) in the study group.

Even though low-moderate penetrance genes are still not candidates for screening in the general population, studies reporting their involvement in BC cases prove that geographical mapping is mandatory since counseling is based on ancestry and ethnicity. Family history appears to be a prerequisite for cancer development and for earlier ages at cancer diagnosis. As such, there are countries where such genes might warrant testing in the future, depending on their reported implication.

At the same time, we must highlight the potential association of low-moderate penetrance genes to factors of poor prognosis in BC patients, including bilaterality, multifocality and triple negativity.

The *CHECK2* mutation has been reported to be associated with bilaterality of BC, a finding also observed in three of our patients—again, on the background of family history and geographical pool [25,26].

In our population, we identified two cases depicting the low-penetrance MSH6 gene mutation who developed multifocal BC. The patient with the c.2136delG variant associated with the *MUTYH* gene mutation c.1187G>A from the insufficient evidence group.

A group of seven patients exhibited overlapping mutations in various combinations between either moderate and high-risk, moderate–moderate risk, moderate–insufficient evidence, moderate VUS and even a patient with a VUS and insufficient evidence gene. This provides more reason to continuously report these cases since penetrance of various genes may be influenced by the ethnical pool, or we may speculate that some gene combinations may potentiate one another, boosting overall penetrance and breast cancer susceptibility.

## 4. Materials and Methods

Our study is a retrospective analysis of 255 patients with BC who presented for oncological examination at the Oncosurg Surgical Oncology Clinic Cluj-Napoca, North-Western Romania, between January 2015 and December 2019 and met the NCCN criteria for genetic testing. One hundred and five patients were excluded due to the negative genetic testing results (Figure 2).

Each patient was approached for genetic testing following BC diagnosis, prior to any other oncological management strategies (chemotherapy or hormonal therapy). Genomic DNA was prepared after sampling 5–10 mL of peripheral blood. Genomic DNA obtained from the submitted sample was enriched for targeted regions of 25 genes involved in hereditary predisposition to cancer: *ATM, BARD1, BLM, BRCA1, BRCA2, BRIP1 (RCA1 Interacting Protein 1), CDH1, CHEK2, FAM175A (Family with sequence similarity 175 member A), MEN1, MLH1, MRE11A, MSH2, MSH6, MUTYH, NBN, PALB2 (Partner and Localizer of BRCA2), PMS2, PTEN (Phosphatase and TENsin homolog deleted on chromosome 10), RAD50, RAD51C, RAD51D, STK11 (serine/threonine kinase 11), TP53, XRCC2* [68].

Sequencing was achieved using the Illumina platform (MiSeq System, San Diego, CA, USA) and followed by orthogonal technologies to confirm clinically significant observations. Orthogonal technologies are used to ensure that variant calls are independently confirmed and thus accurate [12]. All targeted regions were sequenced with a ≥100× depth. This assay targets all coding regions of the indicated transcript, 10 base pairs of flanking intronic sequence, and specific intronic and intragenic genomic regions demonstrated to be causative of disease. Only targeted loci were analyzed for some genes. Large genomic rearrangements were sought by the MLPA technique (Multiplex Ligation—dependent Probe Amplification [50].

The genes were grouped into three risk categories based on penetrance data (Figure 2) [50,65]:

- high-penetrance breast cancer susceptibility genes: *BRCA1, BRCA2, TP53, PALB2, CDH1, STK11, PTEN;*
- moderate-risk genes: *ATM, CHEK2, BARD1, RAD51C, RAD51D, NF1 (Neurofibromatosis type 1);*
- low-risk genes: *MSH2, MSH6, MLH1, PMS2, EPCAM (epithelial cellular adhesion molecule);*
- insufficient evidence: *RAD50, RAD51B, BRIP1, NBN, BLM (Bloom syndrome helicase), FAM175A, MEN1 (multiple endocrine neoplasia 1), MRE11A, MUTYH, XRCC2, APC (adenomatous polyposis coli), RET (rearranged during transfection), FANCA (Fanconi anaemia, complementation group A)*.

There were no novel mutations identified during the study group genotyping.

**Figure 4:**
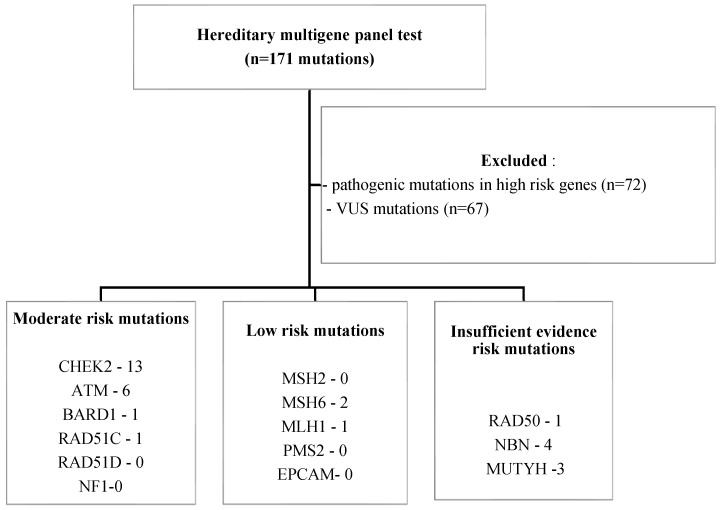
Study population—inclusion and exclusion criteria. (*NF1—Neurofibromatosis type 1, EPCAM—epithelial cellular adhesion molecule*).

High-risk mutation carriers (Table 4) were not included in the present analysis, being considered as exclusion criteria Figure 4.

Seven patients depicted genes of various penetrance associations, as exhibited in Table 5.

## 5. Conclusions

Reporting PVs and VUS across a broader range of genes, including those with limited evidence for BC risk association, is essential for gathering data that may refine risk assessment, guide personalized treatment, and ultimately improve clinical decision making. This approach also enables the development of more targeted BC screening and prevention strategies tailored to the Romanian population’s genetic profile.

## Figures and Tables

**Table 1:** Moderate-risk, low-risk and insufficient evidence risk genes responsible for BC, identified in our study population (VUS—variants of uncertain significance).

	Mutations	Pathogenic Variants		VUS	
		Case Nr	Mutation Type	Case Nr	Mutation Type
Moderate risk	*CHEK2*	13	c.470T>C (p.Ile157Thr) (7 cases) 1283C>T (p.Ser428Phe) c.349A>G (p.Arg117Gly) c.1232G>A (p.Trp411Ter) c.909?-1095 delA c.902delT (p.Leu301fs) c.444+1G>A	1	c.1313A>T
	*ATM*	6	c.7630-2A>C (2 cases) c.2250G>A c.1564_1565delGA c.5318delA c.6628delC	9	c.9077T>G c8734A>G c.3331C>G c.2518G>A c.1444A>C c.2735G>A c.680C>T c.4768C>T c.2735G>A
	*BARD1*	1	c.632T>A	4	c.1333G>A c.2282G>A c.26_40del15 c.1915T>C
	*RAD51C*	1	c.905-2A>G	5	c.790G>A c.1063G>A
	*RAD51D*	-	-	-	-
Low risk	*MSH2*	-	-	1	c.1597C>G
	*MSH6*	2	c.3261dupC c.2136delG	2	c.1068T>G c.2189A>G
	*MLH1*	1	c.2041G>A	-	-
	*PMS2*	-	-	5	c.620G>A c.2012C>T c.852A>G c.46A>G c.2149G>A
Insufficient evidence	*NBN*	4	c.657_661delACAAA (4 cases)	-	-
	*MUTYH*	3	c.721C>T c.536A>G c.1187G>A	-	-
	*RAD50*	1	c.2165dupA	-	-

*CHEK2—Checkpoint kinase 2, ATM—Ataxia-telangiectasia Mutated, BARD1—BRCA1-associated ring domain 1, RAD 51C—Radiation Sensitive 51 Paralog C, MSH—melanocyte-stimulating hormone gene, MLH1—MutL homolog 1, PMS—postmeiotic segregation increased 2, NBN—nibrin, MUTYH—mutY homolog*.

**Table 2:** Pathogenic *CHEK2* mutation variants identified in the study group patients.

HGVS Mutation Nomenclature	Cases (No.)	Percentage (%)	Risk Estimation	Type
c.470T>C (p.Ile157Thr)	7	53.84	Likley Pathogenic	Missense
1283C>T (p.Ser428Phe)	1	7.69	Pathogenic	Missense
c.349A>G (p.Arg117Gly)	1	7.69	Pathogenic	Missense
c.1232G>A (p.Trp411Ter)	1	7.69	Pathogenic	Nonsense
c.909-?_1095+?del	1	7.69	Pathogenic	Deletion
c.902delT (p.Leu301fs)	1	7.69	Pathogenic	Frameshift variant
c.444+1G>A	1	7.69	Pathogenic	Splice donor variant

**Table 3:** Pathogenic *ATM* variants identified in the study group patients.

HGVS Mutation Nomenclature	Cases (No.)	Percentage (%)	Risk Estimation	Type
c.7630-2A>C	2	33.33	Pathogenic	splice acceptor variant
c.2250G>A	1	16.66	Pathogenic	synonymous variant
c.1564_1565delGA	1	16.66	Pathogenic	frameshift variant
c.5318delA	1	16.66	Pathogenic	frameshift variant
c.6628delC	1	16.66	Pathogenic	frameshift variant

**Table 4:** Exclusion criteria for the study group: patients with identified high-risk mutations of the study population [65].

Pathogenic Variants in High Risk Genes	Number of Patients	HGVS Mutation (Human Genome Variation Society)
*BRCA1*	43	c.3607C>T (14 cases) c.5266dupC (11 cases) c.181T>G (3 cases) c.3700_3704delGTAAA (3 cases) c.2241dupC (2 cases) c.843_846delCTCA (2 cases) c.135-2A>G (single case) c.4035delA (single case) c.1789G>A (single case) c.737delT (single case) c.3187C>T (single case) c.4986+6T>C (single case) c.212+1G>T (single case) c.5030_5033delCTAA (single case)
*BRCA2*	21	c.9371A>T (9 cases) c.8755-1G>A (3 cases) c.1528G>T (single case) c.9253delA (single case) c.7007G>C (single case) c.8695C>T (single case) c.7209_7212delCAAAinsGG (single case) c.6557C>A (single case) c.793+1G>A (single case) c.3462delC (single case) c.8655dupA pat (single case)
*TP53*	2	c.469G>T (2 cases)
*PALB2*	6	c.93dupA (3 cases) c.509_510delGA (single case) c.3549C>G (single case)

**Table 5:** Overlapping mutations registry of the study group patients.

**Moderate Risk mutation**	**High Risk Mutation**
*CHEK2* c.470T>C	*BRCA1* c.843_846delCTCA
	
**Moderate Risk mutation**	**Moderate Risk mutation**
*CHEK2* c.444n+1G>A	*ATM* c.7630-2A>C
	
**Moderate Risk mutation**	**Insufficient evidence**
*RAD51C* c.905-2A>G	*MUTYH* c.536A>G
*ATM* c.5318delA	*MUTYH* c.721C>T
	
**Moderate Risk mutation**	**VUS**
*CHEK2* c.1283C>T	*BLM* c.1642C>T
*CHEK2* c.1232G>A	*RAD50* c900G>A
	
**Insufficient evidence**	**VUS**
*NBN* c.657_661delACAAA	*MRE11A* c.1091G>A

## Data Availability

The data presented in this study are available on request from the corresponding author. The data are not publicly available due to restrictions on patient privacy.

## Abbreviations

The following abbreviations are used in this manuscript:

BC	Breast Cancer
HBOC	Hereditary Breast and Ovarian Cancer
NCCN	National Comprehensive Cancer Network
ESO	European School of Oncology
ESMO	European Society for Medical Oncology
HGVS	Human Genome Variation Society
PV	pathogenic variants
ATM	Ataxia Telangiectasia Mutated protein
VUS	variants of uncertain significance
CHECK2	checkpoint kinase 2
LoF	loss-of-function
BARD1	BRCA1-associated RING domain 1
PARP	Poly (ADP-ribose) polymerase
MUTYH	mutY DNA glycosylase
ATM gene	Ataxia-telangiectasia Mutated gene
BARD1	*BRCA1*-associated ring domain 1
RAD51C	Radiation Sensitive 51 Paralog C
MSH1	melanocyte-stimulating hormone gene
MLH1	MutL homolog 1
BRCA	BReast Cancer
TP53	tumor protein p53
PTEN	Phosphatase and TENsin homolog deleted on chromosome 10
PALB	Partner and Localizer of BRCA2
STK11	serine/threonine kinase 11
CDH1	Cadherin 1
PMS2	postmeiotic segregation increased 2
NBN	nibrin
Xrcc	X-ray repair cross-complementing 2
MRE11A	meiotic recombination 11 homolog A
BRIP1	BRCA1 Interacting Protein 1
FAM175A	Family with sequence similarity 175 member A
NF1	Neurofibromatosis type 1
EPCAM	epithelial cellular adhesion molecule
BLM	Bloom syndrome helicase
